# Brain metabolite levels and language abilities in preschool children

**DOI:** 10.1002/brb3.547

**Published:** 2016-08-11

**Authors:** Catherine Lebel, Frank P. MacMaster, Deborah Dewey

**Affiliations:** ^1^ Department of RadiologyUniversity of CalgaryCalgaryABCanada; ^2^ Child & Adolescent Imaging Research (CAIR) ProgramAlberta Children's Hospital Research InstituteUniversity of CalgaryCalgaryABCanada; ^3^ Department of PediatricsUniversity of CalgaryCalgaryABCanada; ^4^ Department of PsychiatryUniversity of CalgaryCalgaryABCanada; ^5^ Mathison Centre for Mental Health Research & EducationHotchkiss Brain InstituteUniversity of CalgaryCalgaryABCanada; ^6^ Strategic Clinical Network for Addictions and Mental HealthAlberta Health ServicesUniversity of CalgaryCalgaryABCanada; ^7^ Department of Community Health SciencesUniversity of CalgaryCalgaryABCanada; ^8^ Owerko Centre at the Alberta Children's Hospital Research InstituteUniversity of CalgaryCalgaryABCanada

**Keywords:** choline, glutamate, language, neuroimaging, phonological processing, spectroscopy

## Abstract

**Introduction:**

Language acquisition occurs rapidly during early childhood and lays the foundation for future reading success. However, little is known about the brain–language relationships in young children. The goal of this study was to investigate relationships between brain metabolites and prereading language abilities in healthy preschool‐aged children.

**Methods:**

Participants were 67 healthy children aged 3.0–5.4 years scanned on a 3T GE MR750w MRI scanner using short echo proton spectroscopy with a voxel placed in the anterior cingulate gyrus (*n* = 56) and/or near the left angular gyrus (*n* = 45). Children completed the NEPSY‐II Phonological Processing and Speeded Naming subtests at the same time as their MRI scan. We calculated glutamate, glutamine, creatine/phosphocreatine, choline, inositol, and NAA concentrations, and correlated these with language skills.

**Results:**

In the anterior cingulate, Phonological Processing Scaled Scores were significantly correlated with glutamate, creatine, and inositol concentrations. In the left angular gyrus, Speeded Naming Combined Scaled Scores showed trend correlations with choline and glutamine concentrations.

**Conclusions:**

For the first time, we demonstrate relationships between brain metabolites and prereading language abilities in young children. Our results show relationships between language and inositol and glutamate that may reflect glial differences underlying language function, and a relationship of language with creatine. The trend between Speeded Naming and choline is consistent with previous research in older children and adults; however, larger sample sizes are needed to confirm whether this relationship is indeed significant in young children. These findings help understand the brain basis of language, and may ultimately lead to earlier and more effective interventions for reading disabilities.

## 1 Introduction

Language skills, including reading precursors such as phonological awareness and speeded naming (Puolakanaho et al., 2007), improve rapidly through the preschool period (Lonigan, Farver, Nakamoto, & Eppe, 2013; Torppa, Poikkeus, Laakso, Eklund, & Lyytinen, 2006). Language difficulties and associated reading problems can have significant negative consequences including poor academic outcomes, mental health problems, and limited career prospects (Goldston et al., 2007; Morris & Turnbull, 2007; Wilson, Deri Armstrong, Furrie, & Walcot, 2009). Much work has been done studying the brain basis of language and reading ability, yet very little is understood about the brain–language relationships in young children, even though language at this time is foundational for the development of future reading skills.

Functional neuroimaging has identified three left hemisphere neural systems critical for reading: two in parietal–temporal and occipital–temporal regions, and one in the inferior frontal gyrus (Kronbichler et al., 2006; Norton, Beach, & Gabrieli, 2014; Paulesu et al., 2001; Shaywitz, 1998; Shaywitz & Shaywitz, 2003). Structural neuroimaging studies show reduced cortical volume, lower white matter integrity, and reduced leftward asymmetry in these regions and the associated white matter connections in children and adults with reading difficulties (Beaulieu et al., 2005; Deutsch et al., 2005; Klingberg et al., 2000; Kronbichler et al., 2008; Lebel & Beaulieu, 2009; Lebel et al., 2013; Nagy, Westerberg, & Klingberg, 2004; Niogi & McCandliss, 2006). Studies examining brain–language relationships in children just beginning to read (aged 5–6 years) suggest that brain alterations are present before reading begins and are not the result of experience‐dependent plasticity (Lebel & Beaulieu, 2009; Raschle, Chang, & Gaab, 2011; Saygin et al., 2013; Vandermosten et al., 2015). A very limited number of studies in preschool children confirm that brain alterations related to language are detectable in preschool children and demonstrate more widespread structure–function relationships than those seen in older children and adults (O'Muircheartaigh et al., 2014; Walton, Frayne, Dewey, Mah & Lebel, 2015).

Magnetic resonance spectroscopy (MRS) accesses biochemical information about metabolite concentrations in vivo, which can help understand normal brain development by providing information about neurons, glia, membrane integrity, and neurotransmitters. MRS has been used to study healthy childhood brain development (Bluml et al., 2013), and increasingly to investigate children with developmental disorders (Baruth, Wall, Patterson, & Port, 2013; Perlov et al., 2009). Brain metabolite concentrations in young children and their relationships with language skills can provide valuable information on the brain metabolism underlying prereading abilities. In addition, such information may assist in understanding the brain metabolites associated with typical language and reading development. However, very few studies have examined relationships between metabolites and reading. One study that measured metabolite concentrations in the occipital cortex in 75 children 6–10 years old with a range of reading abilities, demonstrated negative correlations between composite reading scores and choline/creatine and glutamate/creatine ratios in the occipital cortex (Pugh et al., 2014). Glutamate/creatine ratios also correlated with scores on measures of phonological processing and vocabulary. Similarly, negative correlations between phonological processing and choline/creatine were observed in the left angular gyrus in adults (Bruno, Lu, & Manis, 2013); individuals with better reading skills had lower choline/creatine ratios. In contrast, men with developmental dyslexia were shown to have lower choline/*N*‐acetyl aspartate (NAA) ratios in the left temporal–parietal area compared to normal controls (Rae et al., 1998), though the choice of different baseline metabolites (creatine vs. NAA) makes it difficult to compare findings. Together, these studies suggest that brain choline and glutamate levels play an important role in both reading and language.

Given the relationships between metabolites and reading ability in older children and adults, and evidence showing associations between language ability and brain structure in preschool children before exposure to formal reading instruction, it would be valuable to understand how brain metabolism in young children relates to language abilities that are predictive of later reading. This could help elucidate the roots of reading disabilities, and ultimately help maximize children's potential through early identification and intervention. Therefore, the purpose of this study was to investigate relationships between brain metabolites and prereading language abilities (i.e., phonological awareness, speeded naming) in typically developing preschool‐aged children. Based on previous studies, we hypothesized that glutamate and choline concentrations would be associated with language abilities in young children. To more fully understand brain metabolite–language relationships, we also examined other key metabolites in exploratory analyses. We measured metabolite concentrations in two brain voxels: the anterior cingulate gyrus and the left angular gyrus. These were chosen because the anterior cingulate gyrus is involved in many language tasks (Barch, Braver, Sabb, & Noll, 2000; Chertkow & Murtha, 1997; Marian, Chabal, Bartolotti, Bradley, & Hernandez, 2014; Peelle, McMillan, Moore, Grossman, & Wingfield, 2004; Senhorini et al., 2011), and has been widely studied using MRS in children with developmental disorders (Cochran et al., 2015; Ortiz et al., 2015; Wozniak et al., 2012), although it is not part of the classical reading model. The angular gyrus was chosen as it is a more classical language/reading area and was examined in a previous MRS study of reading in adults (Pugh et al., 2014).

## 2 Methods

### 2.1 Participants

Participants were 67 healthy children with no history of brain disease or trauma, as confirmed via interview questions and a review of medical history. Children were recruited from an ongoing, prospective study that recruited mothers during pregnancy (Kaplan et al., 2014). Parents were asked about history of reading difficulties, children's care settings, and maternal postsecondary education. Nine children had immediate family members with reading difficulties. In terms of child care, 16 children were in day care settings, 34 were cared for by a parent or other relative (typically grandparents), 6 by a nanny/babysitter, and 11 were in combined care (some time in day care, some time at home with a parent). Twenty‐five children were attending preschool part time. Maternal years of postsecondary education was used as a proxy for socioeconomic status, and ranged from 1–13 years (mean: 5.3 ± 3.1 years). All of the children's parents provided informed consent, and all children provided verbal assent before study procedures began. The ethics review board at the University of Calgary approved this study.

**Figure 1:**
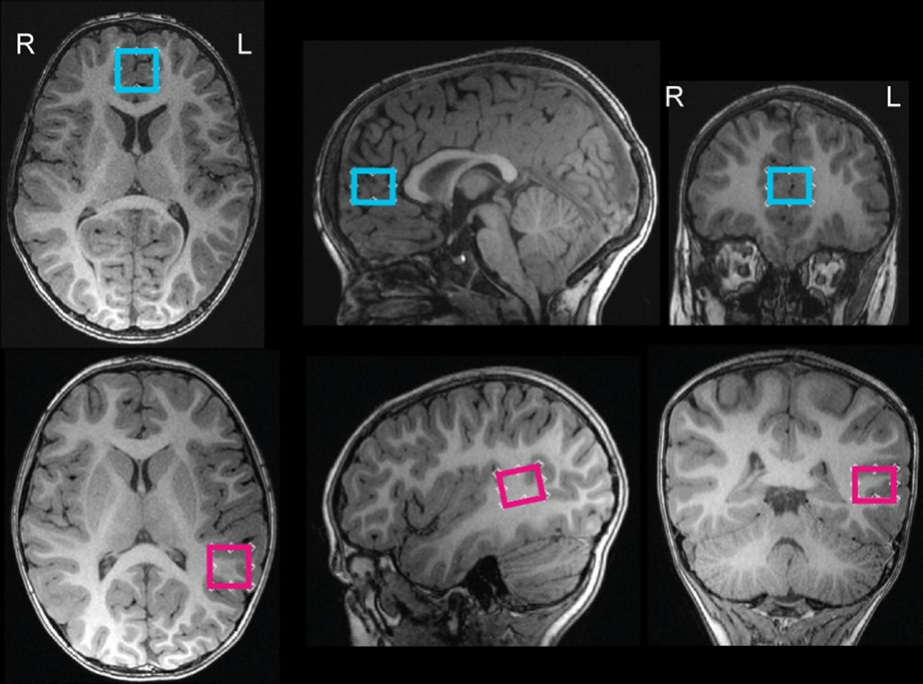
Spectroscopy data were collected from a voxel placed in the anterior cingulate gyrus (top, blue; *n* = 56), or a voxel near the left angular gyrus (bottom, pink; *n* = 45). Due to time constraints, data were only collected from one voxel per imaging session

Twenty‐two children had MRS data collected from a voxel in the anterior cingulate gyrus (Fig. 1), 11 had MRS data collected from a voxel in the left angular gyrus (Fig. 1), and 34 children had MRS data collected from both voxels at separate scanning sessions approximately 6 months apart. Thus, we had data on the anterior cingulate from 56 children (31 males/25 females; age 3.0–4.7 years, 3.5 ± 0.4 years) and data on the left angular gyrus from 45 children (29 males/16 females; age 3.2–5.4 years, 4.0 ± 0.4 years).

### 2.2 Language and cognitive assessments

Language skills were assessed within 1 week of the scan (almost always on the same day) using the NEPSY‐II Phonological Processing and Speeded Naming subtests (Korkman, Kirk, & Kemp, 2007). These tests assess the child's ability to separate different sound components within words (phonological processing), or to rapidly name a series of colors or shapes (speeded naming). Both skills are good predictors of later reading ability (Puolakanaho et al., 2007). Age‐standardized scaled scores were used in the statistical analysis. Higher scores on both subtests indicate better performance (more questions answered correctly for Phonological Processing, faster completion time with higher accuracy for Speeded Naming).

General intellectual assessments were not performed at the time of the MRI scan. However, as part of a separate study on an overlapping group, 44 children were assessed with the Bayley Scales of Infant Development‐III (Bayley, 2005) at 2.5 years of age (mean: 2.5 ± 0.1 years), approximately 1 year before their MRI scan.

### 2.3 Imaging data

Children were scanned at the Alberta Children's Hospital on a 3T GE MR750w MRI scanner. T1‐weighted anatomical imaging was acquired using a spoiled gradient echo sequence with 0.9 × 0.9 × 0.9 mm^3^ spatial resolution, TE/TR = 3.8/8.2 ms. T1‐weighted images were reformatted to provide axial, sagittal, and coronal views, and were used to carefully localize the spectroscopy voxels. Short echo (TE/TR: 30/2000 ms, 128 averages) PRESS proton spectroscopy was conducted with a 15 × 20 × 15 mm voxel placed in the anterior cingulate gyrus or in the left temporal–parietal area (Fig. 1); data were only collected in one voxel per imaging session due to time constraints. The spectroscopy voxel was placed in the anterior cingulate gyrus, anterior to and at approximately the same level as the genu of the corpus callosum as determined on a midsagittal slice. The anterior cingulate gyrus voxel contained almost exclusively gray matter, primarily the rostral anterior cingulate gyrus. The angular gyrus voxel was placed near the left angular gyrus (Fig. 1) using all three imaging planes for accurate localization. It was located in the cortex, excluding as much CSF as possible. This voxel encompassed both white and gray matter, and contained primarily not only the angular gyrus, but also included small parts of the supramarginal gyrus, parietal operculum, and the posterior part of the superior temporal gyrus (Wernicke's area). Both voxels were placed carefully by experienced members of the research team, following illustrations (Fig. 1) and detailed instructions to ensure consistency across participants.

**Figure 2:**
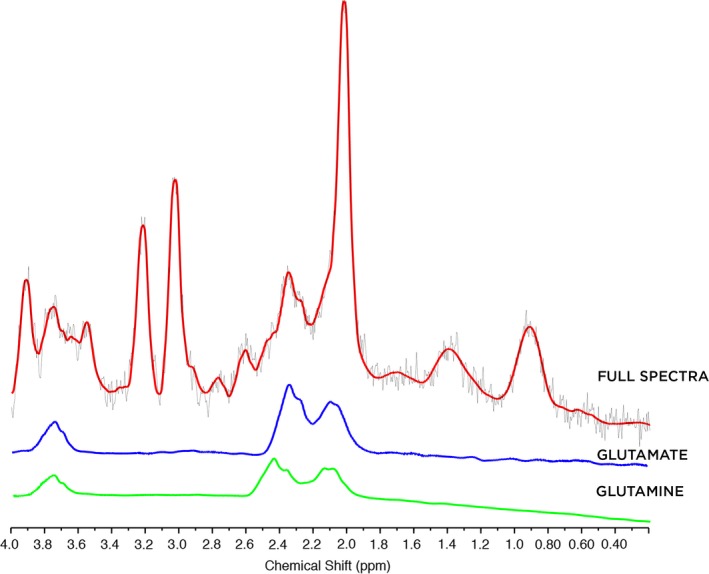
A sample spectrum is shown for a 3.6‐year‐old girl, showing the full spectrum (red) and the separated spectra for glutamate (blue) and glutamine (green)

LCModel (Provencher, 2001) was used to calculate absolute metabolite concentrations (mmol/kg ww) for: glutamate (Glu), glutamine (Gln), creatine + phosphocreatine (Cr/PCr), choline (glycerophosphocholine + phosphocholine), inositol, and NAA. Given the age‐related changes that occur in metabolite concentrations during the preschool years (Bluml et al., 2013; Degnan et al., 2014), we elected to analyze absolute concentrations of metabolites, rather than to normalize to a creatine or NAA baseline, to ensure higher accuracy and interpretability of our findings (Jansen, Backes, Nicolay, & Kooi, 2006). Cramer–Rao bounds were ≤9 for all metabolites except glutamine. More relaxed Cramer–Rao bounds of ≤30 were used for glutamine, as it is more difficult to fit. However, given the very limited reports in the literature on glutamine and its potential to inform future studies, it was included as an exploratory analysis. These bounds for glutamine eliminated three individuals from analysis of the anterior cingulate and one from analysis of the angular gyrus (for glutamine, total *n* = 53 for anterior cingulate, *n* = 44 for angular gyrus). Figure 2 shows a representative spectrum for a 3.6‐year‐old girl, including the isolated spectra for glutamate and glutamine.

Language scores and processed spectroscopy data are available through FigShare: https://dx.doi.org/10.6084/m9.figshare.3381148.v1.

### 2.4 Image/spectra quality

To minimize motion and increase compliance, children were prepared before their MRI scan using child‐friendly explanation of the procedures and in some cases a mock scanner. T1‐weighted images were visually inspected to ensure high quality, and repeated during the scanning session if necessary. Spectroscopy data were originally collected from 70 participants; quality was evaluated using visual inspection of the spectra and Cramer–Rao bounds of the fit provided in LCModel. Based on these criteria, three children had poor‐quality MRS data and were removed from analysis, leaving the full sample of 67 (*n* = 56 with anterior cingulate data; *n* = 45 with angular gyrus data). All T1‐weighted images in these 67 children were of high quality.

### 2.5 Statistical analysis

Analyses were conducted separately for each voxel. Specifically, standardized language scores (Phonological Processing Scaled Score, Speeded Naming Combined Scaled Score) and metabolite concentrations (glutamate, glutamine, creatine/phosphocreatine, choline, inositol, NAA) were tested for normality and homoscedasticity. Following this, language scores were tested for correlations with metabolite concentrations using Spearman skipped correlations (Pernet, Wilcox, & Rousselet, 2012). This method excludes bivariate outliers and provides a more robust estimation of the relationship between variables than typical Spearman correlations. As this is an exploratory study, significance was set at *p* < .05. For comparison purposes to earlier studies, our results from regular Spearman correlations are also reported.

To test whether language–metabolite relationships were specific to language or more generally related to cognitive function, we also examined relationships between Bayley‐III Cognitive Scale scores (at age 2.5 years; approximately 1 year before MRI scanning) and metabolite levels in the children who completed the Bayley‐III (*n* = 37 for anterior cingulate; *n* = 29 for angular gyrus).

Sex differences in metabolite concentrations and language assessments using two sample *t* tests were also investigated, as girls have been reported to have more advanced language skills than boys in the preschool period (Bornstein, Hahn, & Haynes, 2004).

## 3 Results

### 3.1 Language assessments

All 56 children with data in the anterior cingulate completed the NEPSY‐II Phonological Processing subtest, with an average score of 10.2 ± 3.2 (range: 2–16; Table 1). Due to time constraints or refusal to continue, 53 children completed the Speeded Naming subtest, which was conducted after the Phonological Processing subtest. The mean Combined Scaled Score on Speeded Naming was 10.7 ± 2.9 (range 6–19). These scores are not significantly different from the standardized population norms for these subtests of 10 ± 3.

**Table 1:** Demographic information, language scores, and metabolite concentrations are shown for each group of subjects: those with a voxel placed in the anterior cingulate and those with a voxel placed in the left angular gyrus

	Anterior cingulate gyrus (*n* = 56)	Left angular gyrus (*n* = 45)
Age (years)	3.54 ± 0.5	4.03 ± 0.5
Sex	32m/24f	29m/16f
NEPSY‐II Phonological Processing	10.2 ± 3 (*n* = 55)	11.4 ± 3
NEPSY‐II Speeded Naming	10.7 ± 3 (*n* = 52)	12.2 ± 3 (*n* = 44)
Bayley‐III Cognitive Scale	120 ± 16 (*n* = 37)	121 ± 16 (*n* = 29)
Glutamate	11.3 ± 1.1	10.4 ± 1.2
Glutamine	3.8 ± 0.9 (*n* = 53)	5.3 ± 0.8 (*n* = 44)
Choline	1.8 ± 0.3	1.8 ± 0.2
Inositol	5.6 ± 0.7	5.1 ± 0.5
Creatine	6.6 ± 0.8	6.6 ± 0.6
*N*‐acetyl aspartate	8.8 ± 1.1	9.8 ± 0.6

*N* = 56 for the anterior cingulate and 45 for the angular gyrus, unless otherwise noted. Thirty‐four children from each group were the same—the voxel was placed in the anterior cingulate at their first scan and in the left angular gyrus at a follow‐up scan approximately 6 months later.

Similarly, all 45 children with data in the angular gyrus completed the Phonological Processing subtest (mean Scaled Score = 11.4 ± 3.3, range = 3–17; Table 1), while 44 completed the Speeded Naming subtest (mean Combined Scaled Score = 12.2 ± 2.8, range = 4–17). These scores were significantly higher than the standardized population norms of 10 ± 3 for Phonological Processing (*p* = .005) and Speeded Naming (*p* < .001).

Language scores at the second time point (data from the angular gyrus) were higher than scores at the first time point (data from the anterior cingulate). While a practice effect is a possibility, test developers showed only small to negligible practice effects on the Speeded Naming and Phonological Processing subtests (Korkman et al., 2007), and because we are not comparing scores across time, this potential bias is of little concern.

Average Bayley‐III Cognitive Scale scores were 119 ± 16, with a range from 95–145, indicating average to above average functioning in these children. Language and cognitive scores are reported in Table 1 separately for children with data in each voxel.

### 3.2 Correlations between metabolite concentrations and language scores

**Figure 3:**
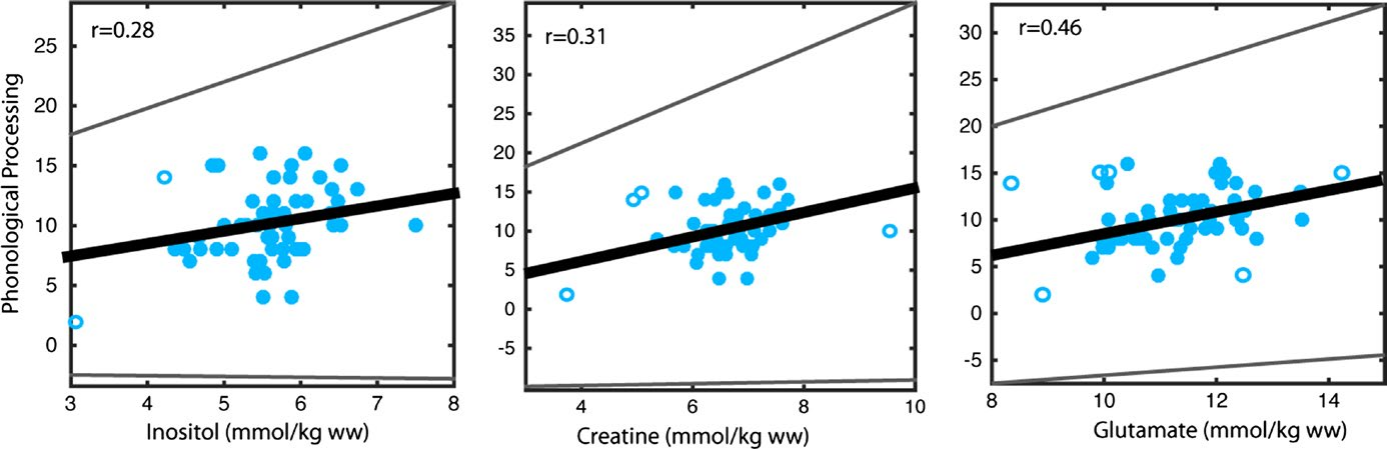
Inositol, glutamate, and creatine in the anterior cingulate gyrus showed positive correlations with Phonological Processing scores such that higher concentrations were associated with better language skills in children (*p* < .05). Bold line indicates best fit, while thinner gray lines indicate the 95% confidence intervals of the correlation. Empty circles indicate bivariate outliers that were not included the skipped correlation analysis

**Figure 4:**
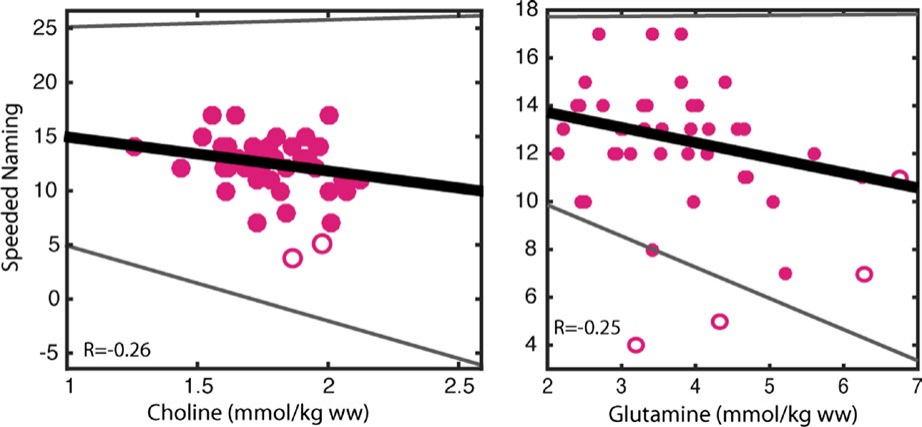
Choline and glutamine concentrations in the left angular gyrus had trend‐level negative correlations with Speeded Naming scores (*p* < .10). Children with lower concentrations of these metabolites tended to have better language skills than those with higher concentrations. Bold line indicates best fit, while thinner gray lines indicate the 95% confidence intervals of the correlation. Empty circles indicate bivariate outliers that were not included the skipped correlation analysis

Creatine and glutamine concentrations in the anterior cingulate gyrus had non‐normal distributions; all other metabolites in the anterior cingulate, and all metabolites in the angular gyrus had normal distributions. All variables showed unequal distributions (heteroscedasticity). In the anterior cingulate gyrus, Phonological Processing scores were significantly positively correlated (skipped Spearman correlations) with creatine (ρ = .31; CI = 0.04–0.54), glutamate (ρ = .28; 0.18–0.67), and inositol (ρ = .27; CI = 0.008–0.54) concentrations (Fig. 3, Table 2). Regular Spearman correlations showed significant correlations between Phonological Processing and glutamate (ρ = .28, *p* = .035) and inositol (ρ = .27, *p* = .043) concentrations; creatine was correlated with Phonological Processing at trend level (ρ = .23, *p* = .087). In the left angular gyrus, there were no significant Spearman skipped correlations, though choline (ρ = −.26; CI = −0.55 to 0.09; *p* = .091) and glutamine (ρ = −.25; CI = −0.55 to 0.09; *p* = .099) displayed trend‐level skipped correlations with Speeded Naming Combined Scaled Scores (Fig. 4, Table 2); regular Spearman correlations revealed significant correlations between Speeded Naming Combined Scaled Scores, and choline (ρ = −.30, *p* = .044) and glutamine (ρ = −.32, *p* = .038) concentrations in the left angular gyrus.

**Table 2:** Spearman skipped correlations between metabolite concentrations and language abilities are shown for each voxel placement group

	Anterior cingulate gyrus (*n* = 56)		Left angular gyrus (*n* = 45)	
	Phonological Processing	Speeded Naming	Phonological Processing	Speeded Naming
Glutamate	.46*	.15	.12	−.07
Glutamine	.16	−.07	−.04	−.25†
Choline	−.09	−.06	−.02	−.26†
Inositol	.28*	.13	−.13	.12
Creatine	.31*	.04	−.12	−.15
*N*‐acetyl aspartate	.16	−.004	.17	−.13

Rho is given for each Spearman correlation. *p < .05, ^†^ *p* < .10.

The Cognitive Scale Scores from the Bayley‐III were not significantly correlated with metabolite concentrations in either voxel. When the metabolite‐language correlations were rerun on only the subset of participants with cognitive scores, only the glutamate‐Phonological Processing correlation in the anterior cingulate remained significant (ρ = .62, CI = 0.37–0.78).

### 3.3 Sex differences

There were no significant differences between boys and girls for metabolite concentrations for either voxel or language assessment scores.

## 4 Discussion

Our results show for the first time that brain metabolite concentrations and language abilities are related in preschool children. Specifically, phonological processing ability was positively associated with glutamate, creatine, and inositol concentrations in the anterior cingulate. Glutamate in the occipital cortex was previously observed to correlate with reading ability in older children, suggesting that the language–glutamate relationship may be stable with age. Creatine and inositol have not been observed to be related to language or reading in older children and adults, suggesting that these relationships may be unique to young children.

Phonological processing and speeded naming are two important components of language ability that are strongly and independently predictive of later reading ability (Carroll & Snowling, 2004; Furnes & Samuelsson, 2010; Puolakanaho et al., 2007). The Phonological Processing subtest of the NEPSY‐II assesses phonemic awareness, a skill requiring listening, identification, and manipulation of sounds. We observed a positive correlation between glutamate, creatine, and inositol concentrations in the anterior cingulate and phonological processing abilities in young children. Although not part of the classical language model, the anterior cingulate has been implicated in phonological processing (Marian et al., 2014; Senhorini et al., 2011) and other language tasks (Barch et al., 2000; Chertkow & Murtha, 1997; Peelle et al., 2004), clearly demonstrating its importance to language function. Glutamate is the most abundant neurotransmitter in the brain and is the brain's primary excitatory neurotransmitter. Glutamate in the occipital cortex was found to have a positive relationship with reading ability in 6–10‐year‐old children (Pugh et al., 2014). While this is in a different brain region, the consistency between our results and those in older children, suggests a stable glutamate–language relationship over time. Inositol is generally thought to be a marker of glial cells, which play a critical role in supporting glutamate neurotransmission. In our sample, glutamate and inositol levels were strongly correlated (*R* = .59, *p* < .001). Thus, the relationships between phonological processing and both glutamate and inositol may reflect this coupled relationship. In the anterior cingulate, glutamate and the BOLD response are linked (Falkenberg, Westerhausen, Specht, & Hugdahl, 2012), and fMRI studies highlight the role of the anterior cingulate in language processing (Barch et al., 2000; Peelle et al., 2004), which may underlie the relationship observed here. Our results support a role of the anterior cingulate in phonological processing, and suggest that its underlying metabolism may be one of the mechanisms that support phonemic awareness.

Creatine concentration was also correlated with phonological processing in the anterior cingulate. Our study measured creatine as a combination of creatine and phosphocreatine, both of which are involved in adenosine triphosphate metabolism. Creatine is often assumed to be stable and used as the baseline in MRS studies, though our results demonstrate a relationship with language ability. Therefore, the negative relationships between glutamate/creatine and choline/creatine ratios and reading ability reported in previous studies (Bruno et al., 2013; Pugh et al., 2014) may reflect higher creatine levels in better readers. However, future research is needed to clarify these relationships.

The Speeded Naming subtest of the NEPSY‐II is designed to assess semantic access to the production of names for colors and shapes, and assesses a separate aspect of prereading language ability than phonological processing. In our study, Speeded Naming scores were not significantly associated with any metabolites, though they were correlated at trend level with glutamine and choline concentrations in the left angular gyrus. Functional imaging studies show this area and the surrounding temporal–parietal region are involved in various aspects of language, including semantic processing, phonological processing, word reading/comprehension, and attention (Seghier, 2013). Further, this region has often been identified as structurally abnormal in poor readers (Frye et al., 2010; Lebel & Beaulieu, 2009; Lebel et al., 2013; Richlan, Kronbichler, & Wimmer, 2013; Vandermosten, Boets, Wouters, & Ghesquiere, 2012). Naming speed in particular has been associated with angular gyrus gray matter volume in adults (He et al., 2013), and with inferior parietal gray matter volume changes over time in school‐aged children (Houston et al., 2014).

Choline is involved in membrane synthesis and breakdown, and is the major precursor for acetylcholine, a neurotransmitter, and was related to reading skills in two previous MRS studies. One found negative phonological decoding‐choline correlations in the left angular gyrus in adults (Bruno et al., 2013), and another found negative choline‐language correlations in the occipital cortex of children aged 6–10 years (Pugh et al., 2014). Though our findings were only at trend level, they suggest a stable choline–reading relationship across ages, even in preschool children who have not yet received formal reading instruction.

Our study has several limitations. First, measures of cognitive development were not collected at the time of MRI scanning and were only available for some participants at 2.5 years of age. Therefore, although there were no significant correlations with cognitive abilities, we cannot be certain that our results are specific to language abilities rather than related to general cognitive ability. Second, only the glutamate‐phonological processing result in the anterior cingulate survived multiple comparison corrections. Based on previous studies, we hypothesized that glutamate and choline would be related to language abilities in young children; our results support the glutamate relationship and provide limited support for choline. We also chose to examine the other metabolites to provide a more complete picture of the brain–language relationships, and found both creatine and inositol were related to language abilities. More studies are necessary, however, to confirm these findings in different populations. Finally, we did not correct for tissue composition in the voxel. Future studies should calculate this and correct for it in order to be more confident in the results.

In conclusion, our results show that relationships between language skills and brain metabolites exist in young preschool‐aged children before formal reading instruction begins. The relationships between phonological processing, and inositol and glutamate in the anterior cingulate cortex may reflect glial differences underlying language function. The inositol and creatine results have not been previously reported in older children or adults and may reflect unique relationships in preschool children. The choline trend relationship with speeded naming agrees well with previous adult studies, suggesting a stable relationship between choline and language ability throughout development though larger studies are needed to confirm this. This information, combined with future studies of different age groups, populations, and brain regions, will help further clarify the brain basis of language abilities in young children and may ultimately lead to earlier and more effective assessments and interventions for children with reading disabilities.

## Funding Information

Canadian Institutes for Health Research (Grant/Award Number: IHD‐134090, MOP‐136797).

## Disclosures

CL's spouse is an employee of General Electric Healthcare. The other authors have no relevant financial disclosures.

## Conflict of Interest

None declared.
