# Brain metabolite levels and language abilities in preschool children

**DOI:** 10.1002/brb3.623

**Published:** 2016-12-19

**Authors:** Catherine Lebel, Frank P. MacMaster, Deborah Dewey

In Lebel, MacMaster, and Dewey (2016), the following errors were published on this article:

(Pugh et al., 2014) was incorrectly cited in the last sentence of the Introduction section on page 2. This should have been: “The angular gyrus was chosen as it is a more classical language/reading area and was examined in a previous MRS study of reading in adults (Bruno et al., 2013).

In the second paragraph of the Discussion section (page 6, top of second column), Pugh et al. found a negative correlation and not a positive one as stated in the article. The subsequent sentence (“While this is in a different brain region, the consistency between our results and those in older children, suggests a stable glutamate–language relationship over time”), which states the results were consistent with the study, is therefore incorrect and should be deleted.

The corrected text should read as:

“Glutamate in the occipital cortex was found to have a negative relationship with reading ability in 6–10‐year‐old children (Pugh et al., 2014). Inositol is generally thought to be a marker of glial cells, which play a critical role in supporting glutamate neurotransmission…”

## References

[brb3623-bib-0001] Lebel, C. , MacMaster, F. P. , & Dewey, D. (2016). Brain metabolite levels and language abilities in preschool children. Brain and Behavior, 6, e00547. 10.1002/brb3.547 PMC5064348 27781150

